# No significant association between patient self-reported non-adherence to antiretrovirals and HIV-tropism: a preliminary analysis

**DOI:** 10.7448/IAS.15.6.18090

**Published:** 2012-11-11

**Authors:** A Ammassari, C Gori, C Pinnetti, P Lorenzini, M Zaccarelli, P Pierro, M Capobianchi, C Perno, A Antinori

**Affiliations:** 1 INMI L. Spallanzani, IRCCS, Rome, Italy; 2 Policlinico Universitario Tor Vergata, Unità di Virologia Molecolare, Rome, Italy

## Abstract

Nonadherence to antiretroviral therapy (ART) may cause virologic failure and disease progression has been associated with switch of viral coreceptor usage from CCR5 to CXCR4. We aimed to assess the association between patient-reported non-adherence and HIV tropism. This is a cross-sectional analysis. HIV-tropism was performed within routine clinical practice either at start of ART or at virological failure. Adherence questionnaire includes: how many times ART has been taken during the last month, missed doses in the last week, timing deviation, refill interruption, drug holidays. Demographics, epidemiological data, HIV and ART history, CD4 and HIVRNA were collected. To assess co-receptor tropism, env V3 genotyping from viremic plasma HIVRNA was performed. For the analysis, dual/mixed viruses were considered as X4. We included 102 individuals: 76% males; median age 42 y (IQR, 37–46); transmission was heterosexual 37%, homosexual 31%, intravenous drug use 29%. Median nadir of CD4 154/mmc (IQR, 53–274), median zenith of HIVRNA 5.26 (4.72–5.70), 46% had AIDS. 124 tropism tests were: 78% R5, 17% X4, 5% dual/mixed. In cases with previous ART, mono/dual ART was found in 26%, median number of regimens was 5 (IQR, 2–10), median time on triple-ART was 54 months (IQR, 0–123) with median time of HIVRNA <50 c/ml of 16 months (IQR, 6.5–34.9). At HIV-tropism, median CD4 and HIV RNA were 321/mmc (IQR, 210–436) and 2.65 (IQR, 2.65–4.91), respectively. Median time between adherence questionnaire and HIV-tropism was 68 days (IQR, 23–116). At adherence questionnaire, median percentage of ART taken during the last month was 100% (IQR, 90–100), 39% reported missed doses in the last week, 40% timing deviation, 7% refill interruption, 17% drug holidays. At univariate analysis, no statistically significant association between non-adherence and dual/mixed-X4 viruses was found (p>0.1). Also gender, age, HIV transmission, AIDS, CD4 nadir, HIVRNA zenith, mono/dual ART, and number of ART regimens were not associated with type of tropism. Only longer time with undetectable HIVRNA before tropism test showed a lower probability of dual/mixed-X4 viruses (OR for each month 0.95; 95% CI 0.90–1.00; p=0.06). No significant association between adherence and HIV-tropism was found in this preliminary analysis. It is possible that patient self-reported adherence is not able to capture nonadherence behaviors that underlie more pronounced viral replication which may be necessary for tropism switch.

